# Retinal Screening in High-Performance Athletes: A Retrospective Analysis of Asymptomatic Peripheral Lesions in Collision and Non-Collision Sports

**DOI:** 10.1186/s40798-025-00869-y

**Published:** 2025-06-11

**Authors:** Nicolas Arej, Hervé Treguer, Chloé Le Cossec, Benjamin Kakona, Nicolas Mandrillon, Vivien Vasseur, Sébastien Le Garrec, Sylvain Blanchard, Sébastien Bruneau, Sophie Bonnin

**Affiliations:** 1 Department of Ophthalmology, Rothschild Foundation Hospital, 29 Rue Manin, 75019 Paris, France; 2 Department of Ophthalmology, Clermont-Ferrand University Hospital, 58 Rue Montalembert, 63000 Clermont-Ferrand, France; 3 Clinical Research Department, Rothschild Foundation Hospital, 29 Rue Manin, 75019 Paris, France; 4 Medical Department, National Institute of Sport Expertise, and Performance, 11 Avenue du Tremblay, 75012 Paris, France; 5 Medical and Scientific Department, Racing 92 Club, 11 Avenue Paul Langevin, 92350 Le Plessis-Robinson, France

## Abstract

**Background:**

Ocular trauma is a frequent concern among athletes, particularly those involved in collision sports. While overt injuries are well-documented, the prevalence of asymptomatic peripheral retinal lesions resulting from repetitive head or ocular trauma remains underexplored. This study aimed to assess the prevalence of these retinal lesions in elite athletes and to evaluate the potential risk associated with participation in collision sports.

**Methods:**

A monocentric retrospective study was conducted at the Rothschild Foundation Hospital (Paris, France), involving 88 professional athletes with an average age of 26 years, predominantly male (80%). All participants underwent comprehensive ophthalmological screening, including fundus examination and ultra-widefield (UWF) retinal imaging of both eyes. Athletes were categorized based on their sport type: collision sports (62%, primarily rugby) and non-collision sports. The efficacy of UWF imaging was compared to dilated fundus examination for detecting peripheral retinal lesions.

**Results:**

Peripheral retinal lesions were significantly more prevalent in collision sport athletes: 40.5% [30.0–51.0%] for rugby, 40.0% [9.6–70.4%] for boxing and 12.5% [0.0–28.7%] for judo, compared to non-collision sport athletes (6.1% [3.0–11.8%]). The diagnostic sensitivity of UWF imaging was relatively low at 45.2% [34.1–56.2%], though it showed high specificity at 93.6% [88.2–99.0%] when compared to dilated fundus examination.

**Conclusion:**

The study highlights a higher prevalence of peripheral retinal lesions in elite athletes engaged in collision sports. These findings emphasize the need for regular ophthalmological evaluations in this population to mitigate potential risks associated with asymptomatic retinal damage.

## Key points

- Collision sports increase the risk of peripheral retinal lesions in top-level athletes.
- Many peripheral retinal lesions are asymptomatic and detectable only through fundus examination.
- Comprehensive ophthalmic evaluation is essential for accurate diagnosis and management of these lesions.

## Introduction

Elite athletes undergo rigorous training regimens and face a heightened risk of various injuries and medical conditions, including ocular manifestations [1]. The repetitive head and ocular trauma experienced during collision sports may predispose athletes not only to overt eye injuries but also to lesions that might remain asymptomatic. Other factors such as increased intraocular pressure, ocular contusions, and sudden changes in acceleration or deceleration may contribute to the development or exacerbation of ocular lesions in athletes [2].

While ocular trauma is a well-recognized consequence of collision sports participation, the prevalence and characteristics of incidental retinal lesions in this population remain underexplored [3]. Peripheral retinal lesions refer to abnormalities located in the outer edges of the retina, the light-sensitive layer at the back of the eye. These lesions encompass a range of conditions, including lattice degeneration (areas of retinal thinning with a crisscrossed appearance), snail-track degeneration (frost-like retinal lesions), pigmentary changes, white without pressure (translucent white areas), atrophy (loss of retinal tissue), condensed vitreous (vitreous traction on the retina), and peripheral hemorrhages. Although many of these lesions are asymptomatic and benign, some may increase the risk of retinal tears or detachment, posing a threat to long-term visual health conditions [4]. Therefore, understanding the prevalence and risk factors associated with these lesions is crucial for optimizing athletes’ ocular care. Additionally, awareness among sports medicine professionals, coaches, and athletes regarding the risks associated with participating in collision sports is crucial for promoting ocular health and implementing preventive strategies [5].

The diagnosis of peripheral retinal lesions in athletes can be challenging due to their location and often asymptomatic nature. While traditional methods such as fundus examination remain essential, advanced imaging techniques such as ultra-widefield retinal imaging (UWF) offer advantages in terms of field of view but may have limitations in sensitivity compared to dilated fundus examination [6]. Therefore, a comprehensive approach combining clinical assessment and imaging modalities is necessary for accurate diagnosis and management of peripheral retinal lesions in athletes.

The purpose of the present study is to estimate the prevalence of peripheral retinal lesions in elite athletes and the excess risk due to the practice of collision sports. Through a thorough investigation of peripheral retinal lesions in elite athletes, this study aims to contribute to the growing body of literature on sports-related ocular health and inform evidence-based strategies for ocular injury prevention and management in athletic populations.

## Methods

This retrospective monocentric study analyzed data extracted from the ophthalmological records of professional athletes evaluated at the Rothschild Foundation Hospital (Paris, France) between September 2022 and July 2023. The study protocol was approved by the institutional ethics committee (approval number: CE_20230523_6_SBN). In this study, sports were classified as collision or non-collision based on the likelihood and intensity of physical impacts involving the head or body, in part using previously established frameworks [7]. Collision sports are defined as activities where athletes are exposed to frequent and forceful impacts, either through direct collision with other players, equipment, or the environment. Examples include rugby, boxing, and judo, which inherently carry a higher risk of head and ocular trauma due to their combative or physical nature. In contrast, non-collision sports, such as swimming, shooting, and table tennis, are characterized by minimal to no physical collision between participants or with equipment, reducing the likelihood of ocular or head trauma. This distinction is essential for understanding the biomechanical risks associated with different sports and their implications for ocular health.

Athletes underwent both dilated fundus examination and UWF retinal imaging using the Optos® California® (Optos PLC, Dunfermline, Scotland) retinal camera. Demographic characteristics such as age, gender, and sport participation details were recorded. Best corrected visual acuity (BCVA) in log MAR and refractive status, including spherical equivalent (SE) in diopters, were also documented for each athlete. A descriptive analysis of the data was performed, including frequencies and percentages for qualitative variables and means with standard deviations for quantitative variables. Missing data for each variable were presented.

The prevalences of peripheral retinal lesions were calculated with a 95% confidence interval. The presence of at least one abnormality on the fundus and/or on Optos® UWF imaging was counted. The analysis was performed at patient level, and the presence of a lesion in at least one of the two eyes was considered. The performance of UWF in detecting peripheral retinal lesions was further evaluated in a subgroup of rugby players, all of whom underwent fundus examination under optimal conditions (using mydriatic eyedrops and a fundus collision lens) by the two same ophthalmologists (SBo, SBr) and UWF imaging in all four gaze directions.

Given that peripheral retinal lesions are known to be more common in myopic eyes, logistic regressions were performed adjusting for the lowest spherical equivalent between the two eyes. Odds ratios were calculated and presented with a 95% confidence interval. Additionally, diagnostic performance (sensitivity and specificity) of UWF imaging compared to dilated fundus examination at the slit lamp was calculated and presented with 95% confidence intervals. All statistical analyses were performed using R software (version 4.3.0).

## Results

A total of 88 top-level athletes were screened, with an average age of 26 years and the majority being men (80%). Among these athletes, 62% were engaged in collision sports, with rugby players comprising the vast majority of participants. On average, the athletes exhibited good visual acuity, with a spherical equivalent close to emmetropia (Table 1).

**Table 1:** Participants’ characteristics

N (%) or mean (SD)			Whole sample (N = 88)
Age (years)			25.8 (6)
Men			70 (80%)
Collision sport			55 (62%)
Sports	Non-collision sports	Badminton	1 (1%)
		Breakdance	1 (1%)
		Fencing	2 (2%)
		Gymnastics	1 (1%)
		Paralympic shooting	1 (1%)
		Pentathlon	8 (9%)
		Shooting sports	13 (15%)
		Swimming	2 (2%)
		Table tennis	3 (3%)
		Trampolining	1 (1%)
		Boxing	5 (6%)
	Collision sports	Judo	8 (9%)
		Rugby	42 (48%)
Visual acuity of the right eye (log MAR)			–0.2 (0.1)
Visual acuity of the left eye (log MAR)			–0.2 (0.1)
Spherical equivalent of the right eye (diopters) *			0 (0.7)
Spherical equivalent of the left eye (diopters) *			0.1 (0.7)

*1 missing data

SD, Standard Deviation; log MAR, Logarithm of the Minimum Angle of Resolution

The prevalence of peripheral retinal lesions was estimated to be 6.1% [3.0–11.8%] in non-collision sport athletes, 40.5% [30.0–51.0%] in rugby player, 40.0% [9.6–70.4%] in boxers and 12.5% [0.0–28.7%] in judo players. Notably, rugby players showed a significantly higher prevalence of peripheral retinal lesions compared to athletes in non-collision sports (Table 2).

**Table 2:** Estimated prevalences of peripheral retinal lesions by type of sport (N = 88 athletes)

Sport	Prevalence [95%CI]
Non-collision (all categories)	6.1% [3.0–11.8%]
Rugby	40.5% [30.0–51.0%]
Boxing	40.0% [9.6–70.4%]
Judo	12.5% [0.0–28.7%]

CI, Confidence Interval

**Fig. 1:**
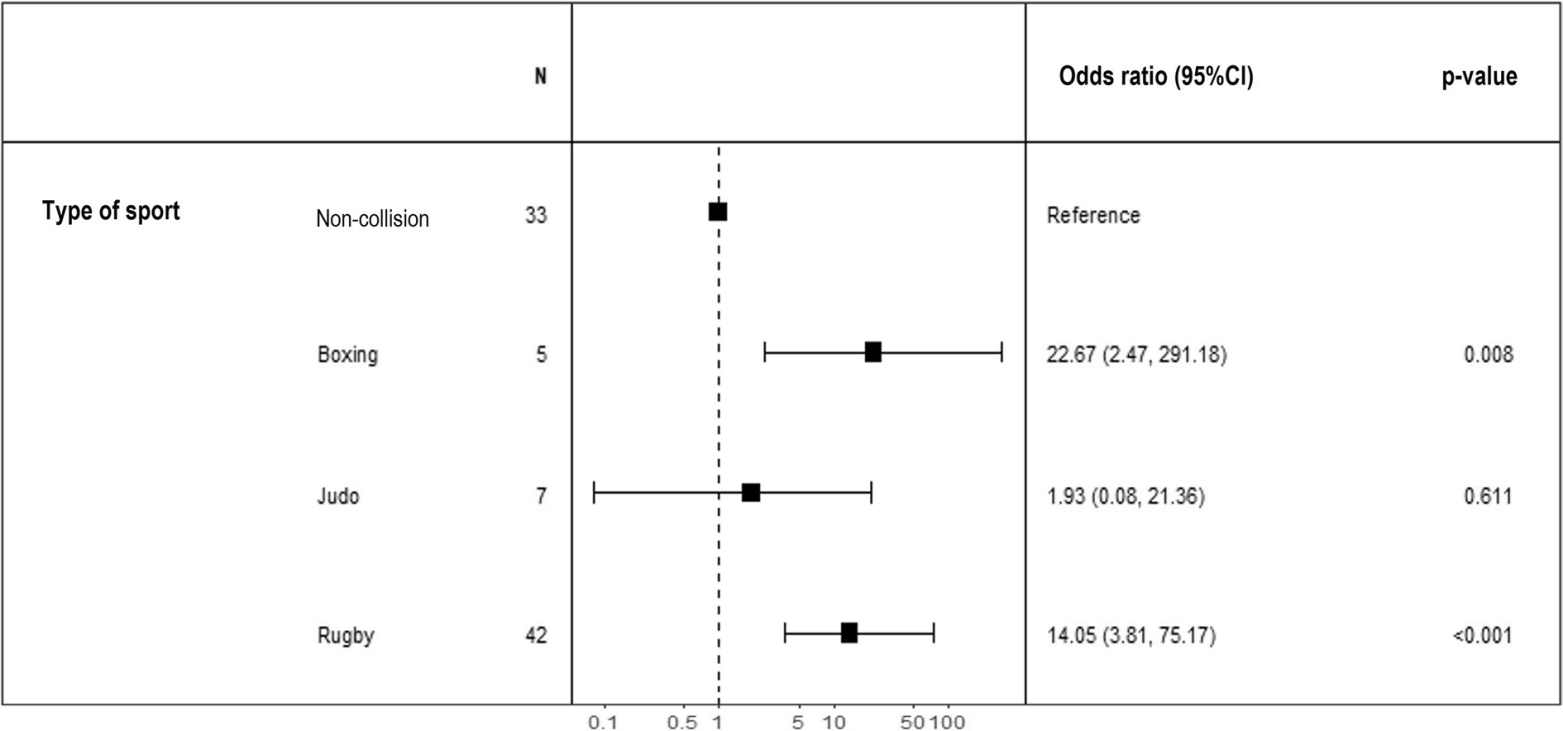
Odds ratio plot, adjusted for the lower spherical equivalent between the two eyes, illustrating the excess risk according to the type of collision sport practiced, non-collision sports of all subtypes being set as a reference (N = 87 athletes). Error bars represent the 95% confidence intervals for each odds ratio

After adjusting for the lower spherical equivalent between the two eyes, participation in collision sports was associated with an increased risk of peripheral retinal lesions in at least one of the two eyes (odds ratio [OR] = 11.82 [2.88—48.52]). Although limited by small numbers, the risk was significantly increased at the 5% threshold for boxing athletes. Additionally, rugby players exhibited a significant excess risk at the 5% threshold of developing a peripheral retinal lesion in at least one eye (Fig. 1).

Among the 55 collision sport athletes, 41 out of 110 eyes (37.3%) exhibited peripheral retinal lesions on fundus examination, while only 25 out of 110 eyes (22.7%) showed lesions on UWF imaging. The detailed distribution of lesions observed by each diagnostic modality is presented in Table 3. It is important to note that one eye may present several different lesions. This was the case for 6 eyes each of which had 2 lesions detected with both fundus examination and UWF imaging. We did not identify any collision sport athlete with more than 2 lesions in the same eye. None of the non-collision sports athletes had more than one lesion in the same eye.

**Table 3:** Types of peripheral retinal lesions and their observed frequencies as identified by fundus examination or Optos® ultra-widefield retinal imaging (UWF) (N = 110 eyes)

	Fundus	UWF
Lattice degeneration	13	4
Pigmentary changes	11	7
Condensed vitreous	6	1
Atrophy	7	1
White without pressure	3	1
Snail-track degeneration	3	0
Hemorrhage	0	1

In the subgroup of the 42 rugby players, 39 players (78 eyes) had all examinations performed under the most optimal conditions. Data from this sample was used to calculate the diagnostic sensitivity of UWF which was 45.2% [34.1–56.2%], with a specificity of 93.6% [88.2–99.0%].

## Discussion

The findings of this retrospective study shed light on the prevalence and characteristics of incidentally discovered peripheral retinal lesions in elite athletes, particularly those engaged in collision sports. The discussion will delve into the implications of these findings, potential mechanisms underlying the observed associations, limitations of the study, and avenues for future research.

### Prevalence of Peripheral Retinal Lesions

The observed prevalence of 36.4% among collision sport athletes is notably higher than previously reported rates in the general population [8], suggesting a potential association between athletic participation and retinal lesions. Notably, boxing and rugby players exhibited a significantly higher prevalence of peripheral retinal lesions compared to athletes in non-collision sports, indicating a potential link between the intensity or nature of sports activity and retinal health. Although most of the eyes were emmetropic or close to emmetropia, adjustment for spherical equivalent helped in reducing the potential bias due to the known association between peripheral retinal lesions and myopia. Myopia is a known risk factor for peripheral retinal lesions due to retinal thinning and stretching, increasing susceptibility to degenerative changes and trauma. While most athletes in this study were near-emmetropic, even a small proportion of highly myopic individuals could have influenced the prevalence of lesions, particularly in collision sports, where trauma-related risks may amplify pre-existing vulnerabilities. To address this, logistic regression models were adjusted for the lowest spherical equivalent (SE) between the two eyes. However, the lack of refractive subgroup analyses (e.g., low, moderate, high myopia) limits the ability to fully isolate the effects of trauma from baseline myopia-related risks. Future studies should stratify participants by refractive status to better distinguish the independent effects of trauma and myopia on peripheral retinal health.

### Risk Factors and Mechanisms

The increased risk of peripheral retinal lesions associated with participation in collision sports, warrants further investigation into the underlying mechanisms. One potential explanation could be the repetitive head and ocular trauma experienced by athletes during collision sports, leading to microtrauma or vascular changes in the peripheral retina. The biomechanical forces exerted on the eyes and surrounding structures during collisions or impacts may predispose athletes to retinal vascular diseases, retinal tears, or other peripheral lesions [9]. Additionally, factors such as increased intraocular pressure, ocular contusions, and sudden changes in acceleration or deceleration may contribute to the development or exacerbation of peripheral retinal lesions in collision sports athletes [10]. Further studies incorporating longitudinal assessments and advanced imaging modalities are needed to elucidate the specific mechanisms underlying the observed associations and to identify potential modifiable risk factors for preventive interventions.

### Pathophysiological Implications

The pathophysiology of peripheral retinal lesions varies by type but typically involves thinning or weakening of retinal tissue, often exacerbated by mechanical stress. Lattice degeneration, for example, manifests as localized retinal thinning with crisscrossed patterns of sclerosed vessels and pigmentary changes, making it susceptible to retinal tears or detachment. In collision sports, repetitive head and ocular trauma amplify shearing forces at the vitreoretinal interface, increasing the risk of microtears or degeneration. [11]. Similarly, snail-track degeneration, a variant of lattice degeneration characterized by frost-like lesions, may result from vitreoretinal traction and stress caused by high-impact activities like rugby or boxing. Other lesions, such as pigmentary changes and white without pressure, may stem from focal ischemia or microtrauma to the retinal pigment epithelium (RPE). These changes are aggravated by acceleration-deceleration forces common in collision sports. Condensed vitreous, driven by repetitive trauma, can create abnormal vitreoretinal adhesions, further increasing the risk of retinal tears. Atrophy of the peripheral retina, linked to ischemic or traumatic processes, may result from transient spikes in intraocular pressure or direct mechanical trauma, compromising retinal microcirculation. Similarly, hemorrhages in the peripheral retina, caused by blunt trauma, may rupture capillaries, leading to localized retinal damage. Over time, repeated hemorrhages can promote degenerative changes. While most peripheral retinal lesions remain asymptomatic and typically do not require immediate treatment, regular monitoring is essential, especially in athletes at higher risk. Follow-up evaluations are critical to detect complications such as retinal tears or detachment, although none were observed in the present study’s population. Longer-term follow-up will be necessary to fully assess these risks.

### Diagnostic Challenges and Clinical Implications

UWF imaging offers a rapid, non-invasive tool that captures up to 200 degrees of the retina, making it ideal for screening. It is notably more cost-effective than traditional dilated fundus examination, especially in large-scale preventive screenings. UWF imaging requires less time, staff, and equipment needed compared to the more time-consuming and resource-intensive process of fundus examination with mydriasis and scleral depression. While traditional fundus exams offer a higher sensitivity for detecting subtle peripheral lesions, UWF’s lower operational costs, combined with its speed and ability to cover a large area, make it a more economical choice for initial screenings and ongoing monitoring.

The diagnostic challenges highlighted by the study, particularly the limited sensitivity of UWF imaging in detecting peripheral retinal lesions compared to dilated fundus examination, emphasize the importance of comprehensive ocular assessment in athletes [12]. The low sensitivity of UWF retinal imaging, observed at 45.2% [34.1%—56.2%] in the subgroup of rugby players examined under optimal conditions, may stem from several factors. First, the peripheral retina’s anatomical challenges, such as its curved surface and variable pigmentation, can limit the ability of UWF imaging to capture fine details compared to dilated fundus examination. Additionally, subtle or small lesions may escape detection due to resolution constraints or image artifacts inherent to the technology. While UWF imaging offers high specificity (93.6% [88.2–99.0%]), making it a valuable tool for confirming findings [13], its low sensitivity suggests it cannot reliably replace traditional dilated fundus examination for comprehensive screening, particularly in high-risk populations like collision sport athletes. Similarly, Khan et al. have found that UWF imaging had a limited interest in detecting treatment-requiring peripheral retinal lesions and 360-degree scleral depressed examination should remain the gold standard [6]. To overcome UWF’s limitations, ensuring proper head alignment and fixation is crucial to reduce distortion. Combining UWF with a thorough fundus exam provides a more accurate and complete assessment, particularly in high-risk cases. Dilated fundus examination with 360-degree scleral depression has a high sensitivity for subtle lesions like lattice degeneration or small tears [14]. However, it is time-intensive, requires significant expertise, and can be uncomfortable for patients.

Optical coherence tomography (OCT) provides high-resolution cross-sectional images but is primarily suited for posterior pole evaluation, with limited application for peripheral abnormalities. Emerging technologies, such as wide-field OCT, may enhance peripheral imaging but are not yet widely available. Clinically, these findings underscore the need for regular ophthalmological evaluation, including dilated fundus examination, in athletes engaged in collision sports. Early detection and monitoring of peripheral retinal lesions are crucial for timely intervention and prevention of potential complications, such as retinal tears and detachments. Additionally, awareness among sports medicine professionals, coaches, and athletes regarding the ocular risks associated with collision sports participation is essential for promoting ocular health and injury prevention strategies [15].

### Preventive Strategies to Mitigate Retinal Risk in Collision Sports

The risk of retinal lesions in collision sports may vary based on individual factors, such as the position played in rugby or specific techniques used in boxing. For instance, forwards in rugby may face higher risks due to frequent head impacts during scrums, while boxing techniques emphasizing head defense may reduce ocular trauma. These differences underscore the need for personalized screening protocols that consider the athlete’s role, exposure to impacts, and history of ocular or head injuries. High-risk athletes, such as those in positions or practices involving repetitive trauma, should undergo more frequent and comprehensive retinal evaluations. Tailoring screening intervals and methods to individual risk profiles can enhance early detection and prevention of retinal damage, optimizing both ocular health and athletic performance.

Given the elevated prevalence of peripheral retinal lesions among athletes in collision sports, implementing preventive measures is essential to protect ocular health [16]. Protective eyewear, designed to shield the eyes from direct impacts, has been shown to significantly reduce the risk of ocular injuries in sports like boxing and rugby. While uptake may be limited by concerns about performance or comfort, advancements in lightweight and sport-specific designs could improve compliance. Additionally, modifications in training practices may help mitigate risk. Emphasizing techniques that reduce head and ocular trauma, such as safer tackling strategies in rugby or improved blocking techniques in boxing, could decrease the frequency and severity of impacts. Education for athletes, coaches, and medical staff on recognizing early signs of ocular injury and the importance of regular ophthalmological evaluations is also critical. These strategies, combined with routine retinal screenings, can minimize the long-term visual risks associated with collision sports participation while maintaining athletic performance.

### Other limitations and Future Directions

Several limitations should be considered when interpreting the findings of this study. The retrospective design and single-center setting may limit the generalizability of the results to other athlete populations or settings. The relatively small sample size, particularly in subgroups like boxing (n = 5), and the uneven distribution of athletes, with rugby players constituting nearly half the sample, could skew the results. The cross-sectional data provided does not allow to evaluate long-term consequences or progression of retinal lesions. All participants were selected from elite-level athletes, which may induce a selection bias and another limitation for generalizability. Moreover, a gender disparity was noted with a majority of male athletes (80%) which did not allow to investigate potential differences in the incidence of retinal lesions between male and female athletes.

These limitations deserve to be addressed by conducting prospective, multicenter studies with larger and more diverse athlete cohorts followed in a longitudinal manner to monitor lesion progression or regression. While the cost-effectiveness of incorporating UWF imaging in routine screenings for elite athletes should be evaluated to optimize resource allocation in sports medicine, longitudinal assessments incorporating advanced imaging techniques, such as wide-field OCT, may provide valuable insights into the progression and prognostic implications of peripheral retinal lesions in elite athletes [17, 18]. Furthermore, investigating potential protective factors, such as ocular protective gear or training modifications, may inform preventive strategies to mitigate the risk of ocular injuries and abnormalities in this population [19].

## Conclusion

In conclusion, the present study highlights the elevated prevalence of peripheral retinal lesions among athletes engaged in collision sports, emphasizing the need for proactive ocular health management. Given the potential long-term visual risks associated with these lesions, routine retinal screening protocols should be implemented, particularly for athletes in high-risk sports like rugby and boxing. UWF imaging provides a practical tool for initial screenings, while traditional dilated fundus examinations remain essential for comprehensive assessments. Sports organizations, coaches, and medical professionals should prioritize education on the ocular risks of collision sports and advocate for regular eye evaluations as part of standard athlete care. Future research should explore individualized screening schedules based on sport type, position, and trauma exposure to further optimize retinal health monitoring and prevention strategies. Taking these steps will safeguard athletes’ vision, ensuring both their health and performance are maintained.

## Data Availability

The data that support the findings of this study are not openly available due to reasons of sensitivity and are available from the corresponding author upon reasonable request.

## Abbreviations

BCVA: Best Corrected Visual Acuity
CI: Confidence Interval
logMAR: Logarithm of the Minimum Angle of Resolution
OCT: Optical Coherence Tomography
OR: Odds Ratio
RPE: Retinal Pigment Epithelium
SD: Standard Deviation
SE: Spherical Equivalent
UWF: Ultra-WideField retinal imaging
